# Ketogenic diets and Ketone suplementation: A strategy for therapeutic intervention

**DOI:** 10.3389/fnut.2022.947567

**Published:** 2022-11-15

**Authors:** Christiaan G. J. Saris, Silvie Timmers

**Affiliations:** ^1^Department of Neurology, Donders Institute for Brain, Cognition and Behaviour, Radboud University Medical Center, Nijmegen, Netherlands; ^2^Radboud Center for Mitochondrial Medicine, Nijmegen, Netherlands; ^3^Department of Human and Animal Physiology, Wageningen University, Wageningen, Netherlands

**Keywords:** nutritional ketosis, ketogenic diet, ketone bodies, precision nutrition, ketone supplementation

## Abstract

Ketogenic diets and orally administered exogenous ketone supplements are strategies to increase serum ketone bodies serving as an alternative energy fuel for high energy demanding tissues, such as the brain, muscles, and the heart. The ketogenic diet is a low-carbohydrate and fat-rich diet, whereas ketone supplements are usually supplied as esters or salts. Nutritional ketosis, defined as serum ketone concentrations of ≥ 0.5 mmol/L, has a fasting-like effect and results in all sorts of metabolic shifts and thereby enhancing the health status. In this review, we thus discuss the different interventions to reach nutritional ketosis, and summarize the effects on heart diseases, epilepsy, mitochondrial diseases, and neurodegenerative disorders. Interest in the proposed therapeutic benefits of nutritional ketosis has been growing the past recent years. The implication of this nutritional intervention is becoming more evident and has shown interesting potential. Mechanistic insights explaining the overall health effects of the ketogenic state, will lead to precision nutrition for the latter diseases.

## The ketogenic diet and its metabolic effect

Caloric restriction and various forms of fasting (intermittent fasting, time restricted eating, periodic fasting) showed extension of lifespan in animals and reduced rates of several diseases, especially metabolic diseases, and cancers (1, 2). Fasting elicits evolutionarily conserved, adaptive cellular responses that reduce free-radical production, improves glucose regulation, increases stress resistance, suppresses inflammation, and causes weight loss. In recent years carbohydrate restriction is gaining popularity and attention. Benefits for general health can be weight loss and improved glucose tolerance, but also better blood pressure control and cholesterol profile (3). It is still unclear whether (neuro) protective effects are associated with an overall reduction in calories (hypocaloric diet) or rather by specific nutrient restriction (diet restriction).

**FIGURE 1:**
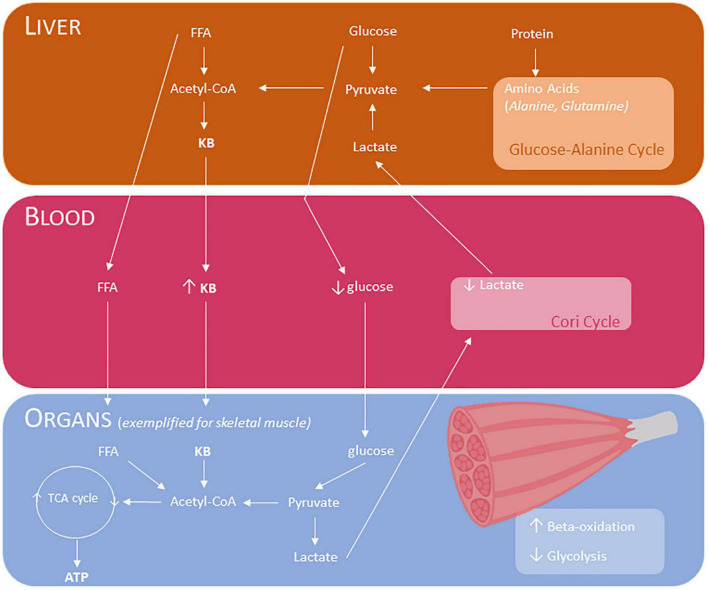
Effects of ketogenic diet and ketone body supplementation and downstream metabolism, exemplified for skeletal muscle. The liver produces ketone bodies during fasting, starvation, in carbohydrate restriction, or prolonged intense exercise. These ketone bodies can be used for input in the TCA cycle in various tissues, including the muscle to generate ATP. The Cori cycle, also known as lactic acid cycle, is the pathway in which lactate is transported to the liver and converted into glucose. Glucose-alanine cycle is the pathway in which alanine is transported to the liver and converted into glucose. KB, ketone bodies (acetoacetic acid/acetoacetate, acetone and beta-hydroxybutyric acid); TG, triglycerides; FFA, free fatty acids; Acetyl CoA, Acetyl coenzyme A; TCA, tricarboxylic acid; ATP, Adenosine triphosphate.

The human body is adapted to survive these periods with no or limited food availability by adapting its substrate preference to sustain bodily functions. During fasting and starvation, but also with carbohydrate restriction, or prolonged intense exercise, the body is stimulating lipid utilization from lipid reserves by mitochondrial beta-oxidation. Whole-body fat oxidation rates increase twofold to threefold (4). The mitochondria of the liver start producing ketone bodies as an alternative fuel source to replace glucose. These ketone bodies (KB), including acetoacetic acid (acetoacetate), acetone and beta-hydroxybutyric acid (βHB), can be utilized by the brain, heart and skeletal muscle through the citric acid cycle (Krebs cycle) and act as an energy source when glucose is not readily available (see Figure 1) (5–7). β-hydroxybutyrate provides more adenosine triphosphate (ATP) per mole of substrate than pyruvate for brain and muscle tissue [for more details on the biochemical signaling, we recommend ref (5)]. Ketosis, referred to by Hans Krebs as “physiological ketosis,” is defined as blood βHB concentrations of ≥ 0.5 mmol/L. The maximum concentration of KB is around 8 mmol/L in undisturbed glucose metabolism while blood pH remains unchanged. Upon refeeding, the presence of insulin and reduction of glucagon will reduce lipolysis and diminish the ketogenetic flux in the liver (8). Besides starvation, also diets low in carbohydrate content can be used to reach a state of ketosis (see Table 1). The latter carbohydrate restricted diets vary in their proportion of carbohydrates. The challenge, however, has been to raise circulating KB levels by using a palatable diet without significant elevated concentrations of plasma cholesterol and free fatty acids (FA). The classic or standard long-chain triglyceride ketogenic diet contains high fat vs. low carbohydrate and low protein content with a 4:1 ratio of fat to protein plus carbohydrate (in grams). Lower ratios can be used to improve adherence. Another strategy to improve adherence is to include days in which more carbohydrates are added to the diet (cyclical ketogenic diet). Targeted ketogenic diet is similar to a standard ketogenic diet except that carbohydrates are consumed just before exercise. Lower ratio with increase in protein content (high protein ketogenic diet) can also be helpful to increase adherence. A very-low-carbohydrate ketogenic diet (VLCKD) is usually referred to as a standard ketogenic diet. Modified Atkins diet (MAD) is adapted from Atkins weight reduction diet in which carbohydrate intake is restricted to 10–20 g/day. Ratio’s between fat to protein plus carbohydrate range typically between 1:1 and 1.5:1, but can reach 4:1. The third type ketogenic diet is the medium-chain triglyceride (MCT) diet. It follows the outline of standard ketogenic diet, but instead of using long-chain triglycerides, medium chain (C6–C12) triglycerides are used since these triglycerides are more ketogenic. Calorie intake is calculated based on the percentage of energy derived from MCT. A fourth strategy is to minimize glycemic increases by higher amounts of carbohydrates with low glycemic index (< 50). This low glycemic index (LGI) diet allows more carbohydrate than either the classic ketogenic diet or the modified Atkins diet and causes more stable glucose levels.

**TABLE 1:** Types of diet to reach nutritional ketosis.

Diet	Specifics	Advantages	Disadvantages
Classic KD, VLCKD	4:1 ratio fat to protein plus carbohydrate	Faster in ketosis, more effective?	Less palatable, difficult to adhere
MAD	Carbohydrate intake restricted to 10–20 g/day (ratio 1:1 to 1.5:1)	More palatable, better adherence	Less effective compared to classic KD?
MCT	Medium chain triglycerides as fat source	MCT are more ketogenic	Not all fat sources can be used.
LGI	Carbohydrates with low glycemic index (<50).	More carbohydrates are allowed	Less production of ketone bodies, less effective?
KB supplementation	Oral ingestion of KB	Better adherence	Only direct effects of KB, no metabolic shift.

Adverse reaction during initiation of the diet as a result of the low glucose levels is termed “keto-flu” and consists of headache, weakness, irritability, constipation, nausea, and vomiting. Symptoms can vary and often diminish after a week of diet. Due to reduction of total calorie intake, since high fat diet is less palatable and fat has a weak effect on satiation, weight loss and anorexia often occur. Polyunsaturated fatty acid intake and reduced intake of cholesterol is important to prevent elevated cholesterol and triglycerides levels associated with atherogenic risk, especially during long-term use of the ketogenic diet (9). Supplementation of omega 3-fatty acids can prevent hyperlipidemia and improve fatty acid profile (10). Gut microbiome alterations in its composition can result in gastrointestinal discomfort, and certain species (Akkermansia, Bacteroidetes, Firmicutes, Muciniphila, Lactobacillus) increase production of short-chain FAs like acetate, propionate and butyrate (11). It is important to avoid malnutrition and include an adequate intake of macro- and micronutrients. Low-carbohydrate diets are often low in thiamin, vitamin B6, folates, vitamin A, vitamin E, calcium, magnesium, iron or vitamin K. There is a reduced bone- and calcium (Ca) metabolism and a risk for developing hypercalciuria and kidney stones (12). Urine analysis and urine calcium creatinine ratio should be analyzed during KD. Good hydration minimizes the risk of stone formation. Mild carnitine depletion occurs in the first months of diet treatment and stabilize or restores slightly with long term treatment (13).

KB can also arise in pathological conditions without carbohydrate restriction. In relative or absolute insulin deficiency, as can occur in diabetic patients, glucose cannot be transported into liver cells, resulting in KB production in the liver (diabetic ketoacidosis). With excessive long-term alcohol consumption an alcoholic ketoacidosis can occur. Hepatic glycogen stores are depleted by malnutrition and ethanol metabolism further impairs gluconeogenesis (14).

In recent years, the effectiveness of exogenous KB supplementation has been explored (15). Oral administered exogenous KB supplementation rapidly elevate plasma levels of KB during a ketogenic diet, but can also be taken as a supplement on top of a normal diet (see Table 1). The metabolic response, due to the presence of glucose, is however different. Exogenous KB lowers fasting glucose concentrations *via* attenuated hepatic glucose output (without increase in skeletal muscle glucose uptake). Dairy products are a natural source of β-hydroxybutyrate with concentration ranging from 10 to 631 μM (16–18). Beta-hydroxybutyrate and acetoacetate salts and/or amino acids, and esters, and MCT supplementation is commercially available and generally considered as safe. Ketone salts, consumed as sodium, potassium and/or calcium βHB, result in high levels of inorganic ion consumption. Ketone esters decrease blood pH and ketone salts increase urinary pH (19). Ketone formulations are racemic mixtures with equal amount of left- and right-handed enantiomers (D-βHB and L-βHB). In the body, D-enantiomers are the predominant circulating KB and is better oxidized than L-enantiomers. It is important to take into account when evaluating these effects of diets or supplementation, that commercial ketone meters and laboratory analysis only detect D-βHB and most do not test for acetoacetate. The combination of KB and MCT supplementation can augment the plasma KB levels to a higher extent and keep them elevated during a more prolonged period (20).

There is no systematical evaluation of cardiovascular risks associated with long term use of KD. Currently, there are no recommendations for routine evaluations such as ECG, cardiac or carotid artery ultrasound (21). Reduced levels of insulin-like growth factor 1 cause growth and bone problems in children. Routine weight and height measurements during KD help to assess growth. Dual energy X-ray absorptiometry (DEXA) and vitamin D as routine examination is recommended during KD and calcium and vitamin D supplementation should be maximized and exercise strongly encouraged. When growth retardation occurs, protein intake should be maximized.

Physical performance and fatigue are dependent on substrate availability to working muscles. As exercise intensity increases, there is a shift in the contribution of different substrates to energy provision from free fatty acids and glucose in the blood toward intramuscular triglycerides and glycogen. At moderate-to high intensity exercise (> 75% of maximal oxygen uptake, VO2max), muscle glycogen is the main energy source (22). Hence, much focus has been directed toward advancing nutrition strategies that might spare glycogen reserves as this could support or even optimize sports performance (23–25).

KB have the potential to be used as an alternative fuel source to carbohydrates and fat during endurance exercise and can lower the exercise-induced rise in plasma lactate (see Figure 1) (26), amongst other metabolic effects (6). Moreover, the capacity to take up and utilize KB during exercise seems higher in exercise-trained skeletal muscle (27).

The potential ergogenic effects of KB supplementation has therefore attracted a lot of scientific interest. Habitual consumption of a ketogenic diet, which is high in fat (∼80% total kcal), very low in carbohydrate (∼5% total kcal), and moderate in protein (∼15% total kcal), augments circulating KB and enhances fat oxidation while sparing carbohydrate oxidation during endurance exercise (28–30). Yet benefits of ketogenic dieting for performance remain equivocal, possibly attributed to a low muscle glycogen content at the start of exercise due to severe carbohydrate restriction and impaired glycolytic flux during high-intensity exercise (31–33). This has led to the exploration of means to achieve acute nutritional ketosis that bypass the undesirable effects of carbohydrate restriction. Acute exogenous ingestion of KB in the form of βHB salts or ketone esters has sparked the interest to enhance performance and recovery in athletes (34, 35). The latter ketone supplements can induce acute ketosis as evidenced by > 0.5 mM βHB in the blood for up to 3 h after consumption of an acute dose, without the necessity to modify dietary intake (19, 36). Hence, they could serve as an attractive strategy to supply the working muscle with extra energy, while sparing muscle glycogen stores (35). In addition, ketone bodies are in essence a more efficient energy substrate than glucose or fatty acids because its conversion into an oxidizable form does not require ATP, thereby enabling more muscle work for a given energy cost (34).

From a performance point of view, the effect of exogenous KB supplementation as an ergogenic aid, especially in the acute form, is not ambiguous. A recent meta-analysis (13 studies) (37) and systematic review (10 studies) (38) both failed to show consistent effects of exogenous KB supplements on physical performance (both endurance and power outcomes). Except for the seminal study of Cox et al. (39), the remainder of the studies failed to show any benefit of acute KB supplementation on performance (40–46), and some even reported detrimental effects (47, 48). Also, on secondary outcome measures like plasma lactate and glucose, respiratory exchange ratio, or perceived exertion the efficacy of KB supplementation remains inconclusive.

The various KB supplements in combination with their pharmacokinetics as well as the variation in gastrointestinal distress between supplements is likely to affect the performance outcome measures. That is, the type of supplement (ketone ester vs. ketone salt) and the nutritional status (fed vs. fasted state) seems to determine the level of circulating βHB concentrations, and hence explain a lot of the variance between studies (37, 38). Providing ketone monoesters in the fasted state maximizes the state of ketosis, reaching peak circulating βHB concentrations above 2 mM (19, 39, 40, 49), which are blunted in the fed state (19). Ingestion of ketone salts (46, 48), ketone diesters (47), ketone precursors (44, 45), or ketone monoesters (41) in the fed state induce acute ketosis (βHB > 0.5 mM), yet fail to reach the 2 mM cutoff. The difference in peak βHB concentrations between ester and salt supplements are likely mediated by the βHB isoform present in the drinks. In contrast to ketone esters, which are composed of D-βHB, ketone salts are often a racemic mixture of both D-βHB and L-βHB isoforms of βHB, even though the metabolism and metabolic fate of L-βHB being less well understood (50, 51). The L-βHB isoform seems less readily oxidized and does not seem to contribute a lot to energy supply, and hence may accumulate upon repeated ketone salt drinks (19). Furthermore, it is documented that food in the gut can delay or prevent the uptake of small hydrophilic carbons such as βHB (52, 53), probably explaining the lower circulating βHB following food consumption (19). KB supplementation is also associated with symptoms of gastrointestinal distress, which can range in severity. Symptoms including nausea, diarrhea, constipation, vomiting and abdominal pain have been reported (36). Another factor that may likely influence the performance benefit is the type of test chosen. As KB have been suggested to exert antiglycolytic effects (39), KB may especially work advantageous during low-to-moderate intensity endurance exercise (35). That is, KDs have shown to be beneficial during a long duration (≥ 12 h), low-intensity ultra-endurance-type event, where mitochondrial beta-oxidation can match lower rates of ATP demand, whereas the combination of nutritional ketosis and high carbohydrate intake may confer performance benefits in endurance events of moderate intensity and durations of ≥ 8 h (54). In summary, even though there is a clear biological rationale to support a performance enhancing effect of acute exogenous KB supplementation, to date there is not sufficient evidence to support a clear ergogenic effect. Due to divergent effect across subjects, this might also suggest responders and non-responders (55).

Overall, nutritional ketosis seems an interesting and promising strategy to increase the health status of healthy individuals and to serve as a therapeutic intervention in several diseases. Insights in these mechanisms will lead to precision nutrition for these diseases (56). Below, the effects on KB on epilepsy, heart diseases, mitochondrial diseases, and neurodegenerative disorders will be summarized.

## Ketogenic diet in (intractable) epilepsy

KD is a well-established, effective non-pharmacologic treatment for intractable childhood epilepsy. Its history dates back to 500 years BC when fasting was already was recognized as an effective therapy against epilepsy; a finding that was also mentioned in Hippocrates Collection, Hippocrates’ medical theories on the treatment of various illnesses (57). Since then, many different diets have passed the revue and in the recent years, KD has gained more popularity in both pediatric and adult patients. Both the classic and the modified KD are effective for the treatment of (Intractable) childhood epilepsy (58, 59). Furthermore, both carbohydrate restricted diets as well as calorie restriction reduce seizures (60) since the reduction of glycolysis is a key element to suppress seizures (61). In a randomized controlled trial (RCT) in children with refractory epilepsy, KD was compared to MAD and LGI (62). All 3 diets showed a similar reduction in seizures (66% for the KD, 45% for the MAD, and 54% for the LGI). While LGI showed relatively fewer adverse events, neither the MAD nor LGI were non-inferior to KD. Of notice is that seizure decline was rapid for KD and MAD, while more gradual for LGI.

An important antiepileptic effect of KD occurs through direct inhibition of receptors by FAs. One of the antiepileptic drugs that interacts with sodium channels, valproic acid, is actually a branched short-chain fatty acid made from valeric acid. Antiepileptic action of a number of PUFAs was also observed on isolated hippocampal neurons. Decanoic acid, an MCT, can directly and selectively inhibit AMPA receptors non-competitively in animal models (63). Decanoic acid is also a peroxisome proliferator activated receptor (PPAR) γ agonist, which elicits neuronal mitochondrial biogenesis (64–66).

Many hypotheses for indirect metabolic antiepileptic effect of KD in the brain have been postulated as well. Increasing ATP availability, reducing reactive oxygen species (ROS) generation by mitochondrial complex I, inhibiting the mitochondrial permeability transition pore, and stimulating mitochondrial biogenesis, all seem to stabilize synaptic functions. That is, βHB can be produced in astrocytes and is metabolized in the mitochondria of all brain cell types (67). KB serve as an efficient mitochondrial fuel, where it can alter the NAD+/NADH and Q/QH2 couples and reduce production of mitochondrial ROS. Evidence for mitochondrial dysfunction in acquired epilepsy comes from the observation that metabolic and bioenergetic changes occur following acute seizures and during different phases of chronic epilepsy (68). Stimulation of mitochondrial biogenesis is important to improve and stabilize synaptic transmission, for which about 30% of the energy in the human brain is spent (69). Ketogenic diet causes a reduction of glycolytically derived lactate in the brain due to a better tolerance to *in vivo* hypoxia (70). Other indirect ways to stimulate the antiepileptic effects of KD may lay in the alterations of the metabolism of neurotransmitters such as glutamate and gamma-amino butyric acid (GABA), and the activation of energy-sensing signaling pathways such as the PPAR, mammalian target of rapamycin (mTOR), and adenosine monophosphate-(AMP)-activated kinase (AMPK) pathways (71, 72). Indeed, KB are able to change GABA and glutamate aminoacid metabolism thereby inhibiting glutamatergic excitatory transmission and mitigate neuronal hyperexcitability (73). These effects were also noted after brain injury in rats with an age-dependent decreased cortical contusion volume with ketogenic neuroprotection (74).

Another system in which KD has influence on is the gut-microbiome (75). Mice treated with antibiotics or reared germ free are resistant to KD-mediated seizure protection, whereas enrichment of *Akkermansia muciniphila* and *Parabacteroides* populations restores anti-epileptic effect of KD (76). Efficacy of KD in seizure reduction in children with refractory epilepsy was associated with changes in intestinal microbiota and specific microbiomes may serve as an efficacy biomarker (77). Addition of probiotics can reduce fat accumulation in the liver caused by ketogenic diet in rats (78).

Intestinal dysbiosis might also be a possible etiopathogenic factor in drug-resistant epilepsy (79). Gut bacteria can release neuropeptides and neurotransmitters, such as serotonin, GABA and glutamate, or their precursors (tryptophan and its metabolites) (80). They are also important for the biosynthesis of short-chain fatty acids. An additional mechanistical connection between the gut and brain is the stimulation of afferent neurons of the enteric nervous system (ENS) by bacteria. Enteric afferent neurons communicate intestinal conditions to the brain through the vagus nerve.

In drug-resistant epilepsy, KD has shown anti-epileptic effect and has a major benefit compared with standard epilepsy treatment with AEDs in children and adolescents due to less long-term adverse effects (81). A meta-analysis showed that treatment with KD in refractory epilepsy in children gives a 5.6 times more likely chance than the control group to have a 50% reduction of seizures after three months of the diet or earlier (82).

Even in infants < 2 years of age, KD can be considered as a non-pharmacological treatment and is currently used in infants with refractory epilepsy syndromes (83). In super-refractory status epilepticus (status epilepticus that persists or recurs 24 h after anesthetic therapy onset or after its withdrawal) KD is recommended as an alternative therapeutic strategy (84). Evidence for KD in refractory epilepsy or status epilepticus in adults is limited, with only one RCT. In comparison with a control group, cotreatment with MAD in adults with refractory epilepsy decreased seizure frequency 2.19 times (85, 86). Effect of KD in epilepsy is thus well-established and has been used for centuries. However, the quality of evidence is low due to the limited number of randomized controlled studies, small sample sizes and the limited studies in adults (87). Large-scale RCTs, refinement of more palatable diets and other nutritional ketogenic strategies are needed to optimize nutritional ketosis in clinical practice as a non-pharmacological treatment strategy in epilepsy.

## Ketogenic diet in cardiology

Energy source of heart tissue is 60–90% oxidation of fatty acids, 10–30% of glucose, and 5% of KB of total ATP under normal conditions, respectively. Per unit mass, the myocardium consumes most of the KB (88, 89). Under hemodynamic stress the ventricles of the heart secrete B natriuretic peptide (BNP) to stimulate the release of free fatty acids from adipose tissue and ketogenesis in the liver to provide heart muscle tissue an additional energy source (90). Atrial natriuretic peptide (ANP) exert potent lipolytic effects by activating hormone-sensitive lipase in adipocytes, however the role of ANP in ketogenesis remains unknown (91).

In the hypertrophied and failing heart, decreased myocardial capacity for fatty acid oxidation occurs, leading to enhanced cardiac glucose utilization through anaerobic glycolysis, pyruvate accumulation and lactate production. This remodeling of mitochondrial energy metabolism is suggested to be a fetal shift resulting in an increase in cardiac efficiency (92). Intramyocardial lipid accumulation and myocardial insulin resistance occur. KBs are able to provide an additional energy source and may therefore improve energetics in the failing heart.

In a mouse model were pressure overload-induced heart failure was established by transverse aortic constriction, continuous KD showed no significant effects on cardiac systolic function and fibrosis but aggravated cardiac diastolic function (93). In the same study, an alternate-day KD, however, exerted potent cardioprotective effects against heart failure after 8 weeks of diet. Continuous KD for 8 weeks also transiently increased endothelial cell proliferation in the heart and prevents capillary rarefication in these mice (94).

A new class of treatments in chronic heart failure are sodium-glucose cotransporter-2 (SGLT2) inhibitors. SGLT2 inhibitors were initially designed as antidiabetic drugs by blocking glucose reabsorption in the proximal renal tubules. As a result, glucose renal excretion will be increased, thereby lowering serum glucose levels and stimulating ketogenesis. These KB improve cardiac energy supply and reduce pathological ventricular remodeling, and inflammation in patients with heart failure (95). Animal studies further support the improvement of heart function in ketogenic therapy. In type 2 diabetic mice, KD reduces mitochondrial fission and hence improves mitochondrial function in the heart (96). Rats fed for 5 days with a ketone ester supplementation diet improved running performance on a motorized treadmill (97). The isolated hearts from these rats, treated with KB supplementation for 66 days, had greater free energy available from ATP hydrolysis during increased work. In another study on rat hearts, addition of both insulin and KB to isolated perfused working rat hearts led to a 35% increased efficiency of cardiac hydraulic work (98).

Besides KB, the KD can also provide a significant source of polyunsaturated fatty acids (PUFAs). These PUFAs can inhibit voltage-regulated sodium and calcium currents, thereby exerting an antiarrhythmic action on the heart (99, 100). In addition, KD lowers heart rate and increases heart rate variability due to reduced sympathetic activity (101).

However, long-term use of KB might promote side effects in the heart. When treated for 16 weeks, KD-fed rats showed increased heart rates and impaired cardiac function. Additionally, increased cardiac fibrosis was found, potentially mediated by βHB-induced effects on the Sirtuin 7 promotor (102).

In summary, nutritional ketosis may serve as a cardioprotective alternative energy supply in the failing heart and reduce pathological ventricular remodeling and could serve as a supportive, non-pharmacologic treatment in people with heart failure along current treatments, such as SGLT2 inhibitors (103, 104). Long-term use of ketosis should however be tested for safety.

## Ketogenic diet in mitochondrial diseases

Mitochondrial diseases are systemic and heterogenic diseases caused by underlying pathogenic variants in nuclear or mitochondrial DNA (mtDNA). Oxidative phosphorylation and ATP production are impaired and energy demanding organs most often show symptoms. Reason for initiation of KD in patients with a mitochondrial disease is intractable epilepsy and similar positive effects on epilepsy compared to patients without a primary mitochondrial disease (105). Few patients have been treated with KD for other reasons (muscle involvement, movement disorder, and intellectual disability) with some reports on improvement of clinical symptoms.

In mitochondrial dysfunction, increased anaerobic glycolysis causes excess amounts of pyruvate that accumulate and is converted into lactate. To prevent acidification of the cell, pyruvate is also transaminated into alanine and lactate is transported out of the cell thereby acidifying the extracellular environment. Lactate and alanine are transported to the liver to be converted again into glucose (gluconeogenesis), *via* the Cori cycle and the glucose-alaninecycle, respectively (see Figure 1). When pyruvate is converted into acetyl coenzyme A, this step is irreversible and therefore carbohydrates can be converted into fats but not vice versa (106).

KD and KB are capable to increase mitochondrial respiration *via* an increase in ATP production and improve the efficiency of the mitochondrial respiratory chain complex with increased mitochondrial biogenesis (69). βHB also reduces the production of ROS thereby improving mitochondrial respiration and bypassing the complex I dysfunction (67, 107). After oxidative stress in neocortical neurons, KB are neuroprotective by inducing expression of mitochondrial uncoupling proteins (UCP) which reduce ROS production (69, 108). In acidic conditions and high levels of ATP and NADH, the mitochondrial membrane permeability transition is further reduced (109). In brown adipose tissue of mice, mitochondrial biogenesis and UCP1 expression was shown to increase with a ketone ester diet (110). Increase in antioxidant activity was accomplished during KD by an increase of glutathione and glutathione peroxidase activity (111, 112). In addition, rats given a diet including decanoic acid-containing triglycerides had increased brain mitochondrial function and ATP synthesis capacity (113). In MCT-fed aged rats mitochondrial density and function in cerebellar Purkinje cells were significantly increased and age-related mitochondrial dysfunction was recovered (114). Also, a mouse model for late-onset mitochondrial myopathy due to overexpression of mutated Twinkle protein, a nuclear-encoded replicative helicase of mtDNA, showed clear health improvements after 10 months of KD. That is, markers for disease progression improved in these mice (115). The amount of cytochrome c oxidase negative muscle fibers in treated mice decreased by 30%, the citrate synthase activity in muscle as a marker of mitochondrial biogenesis was doubled, liver lipid levels were restored and muscle mitochondrial ultrastructure was normalized (no ragged red fiber-like muscle fibers or mitochondria with distorted structure and cristae on electron microscopy).

Few trials have been carried out in patients with mitochondrial myopathy, or on patient-derived material. In five patients with mtDNA deletions with a progressive external ophtalmoplegia phenotype (PEO), the MAD induced progressive muscle pain and muscle fiber necrosis after 2 weeks of diet (116). Muscle biopsy showed lysis of muscle fibers with the most mitochondrial abnormalities (ragged-red fibers). Transcriptomic analysis showed increase in mitochondrial biogenesis, and oxidative phosphorylation. Follow-up analysis after 2, 5 years suggested that muscle regeneration and mild improvement in muscle strength occurred. Lysis of ragged-red fibers might actually be beneficial by inducing satellite cell fusion. In another study, cell cultures of patients with PEO/Kearns-Sayre syndrome grown in a medium containing KB, hence replacing glucose as the carbon source, showed reduced heteroplasmy mtDNA mutation levels (117).

In summary, the above experiments suggest that nutritional ketosis can positively impact on mitochondrial bioenergetics, mitochondrial ROS/redox metabolism and mitochondrial dynamics (118). In human trials, however, the risk for muscle fiber necrosis should be monitored carefully and explored further.

## Ketogenic diet in neurodegenerative diseases

Central features of neurodegenerative diseases are neuronal oxidative stress and mitochondrial dysfunction. The brain hypometabolism in neurodegenerative diseases is associated with reduced glucose utilization as detected by fluorodeoxyglucose positron emission tomography (FDG-PET). The brain preferentially uses KB over glucose (119, 120). A ketogenic diet is able to increase KB substrate and thereby increase brain phosphocreatine (PCr) levels (121). In addition, the KD is able to abate apoptosis of neurons (122).

It is unclear whether neuroprotective properties arise from the combination of elevated concentration of KB with low availability of carbohydrates, or solely from elevated concentration of KB. Hence, it is plausible that caloric restriction is already sufficient for neuroprotection (123, 124). KD and caloric restriction both result in reduction of blood glucose and a reduced glycolytic flux. The restriction of calories will improve mitochondrial functions, leading to reduced ROS production and increased energy output. Both caloric restriction and KD decrease inflammatory and pro-apoptotic activities through activation of PPAR (125, 126). It has also been shown in numerous species, including primates, that caloric restriction prolongs the lifespan (127, 128).

From a brain-function point-of-view, ingesting a single MCT meal improves task performance in healthy elderly. This was accompanied by decreased regional blood flow in the dorsolateral prefrontal cortex, the brain region responsible for executive functions, indicative of extra energy source availability (129). Decanoic acid is able to modulate astrocyte metabolism directly, leading to activation of the astrocyte-neuron lactate shuttle and providing fuel to neighboring neurons in the form of lactate (130, 131). Healthy participants between 55 and 80 years had no effects on cognitive function with supplementation of 30 g MCT for a period of 2 weeks (132).

Most trials in neurodegenerative diseases on KB and KD have been investigated in Alzheimer’s (AD) and Parkinson’s disease (PD).

### Alzheimer’s disease

There is growing evidence that the KD may be an effective treatment for AD largely through enhanced mitochondrial functioning, however, clinical studies to date have been equivocal (133–135). Consumption of monounsaturated, polyunsaturated and omega 3 fatty acids is related to a decreased risk for AD (136). A direct effect of PUFAs is blocking voltage-gated sodium and calcium channels (137). On the contrary, intake of high saturated fat may possibly be associated with an increased risk in AD (138). Also, high glycemic diet can deteriorate the brain, as 1 year of high glycemic dieting was related to precuneal amyloid accumulation in the lateral temporal lobe and posterior cingulate gyrus (139).

In a mouse model of AD, long-term administration of ketone esters lessened amyloid β-peptide and hyperphosphorylated tau deposition, which decreased levels of anxiety and improved cognition (140). The KD was also found to reduce the volumes of soluble amyloid-beta in homogenates of murine brains (141). Four months of KD improved spatial learning, spatial memory and working memory in a mouse model of AD (the 5XFAD mic) (142). At a histopathological level, reduced amyloid plaque deposition and microglial activation was seen, resulting in reduced neuroinflammation. Initiation at a late stage showed no effect on cognitive improvement.

In patients with mild cognitive impairment, 6 weeks of very low carbohydrate diet improved verbal memory performance (143). Urinary ketone levels were positively correlated with memory performance. Consumption of 56 g/day of MCT supplementation for 24 weeks also increased serum KB concentrations and improved memory in subjects with mild cognitive impairment (144). A single oral dosage of MCT led to the elevation of plasma KB levels in AD patients, and to increased cognitive performance for Apolipoprotein E (APOE) ε4 negative, but not for APOE ε4 positive subjects (120). Apolipoproteins are involved in the metabolism of fats, and the epsilon subunit is associated with risk for Alzheimer’s disease. APOE-ε4 status may possibly influence βHB consumption efficacy and may therefore be efficient in APOE ε4 negative patients. A randomized placebo controlled trial in which MCT was given to subjects with mild and moderate AD for 90 days increased serum concentrations of KB and improved cognitive functioning (145). Effects were again most notable in APOE ε4 negative patients who were dosage compliant. In a study in Japan MCT was administered to 20 Japanese patients with mild-to-moderate AD (146). At 12 weeks they showed significant improvement in the digit-symbol coding test and immediate logical memory tests compared to the baseline.

In the Ketogenic Diet Retention and Feasibility Trial, 15 patients with AD maintained an MCT-supplemented KD for 3 months (147). They observed that upon achieving complete ketosis, the mean of the Alzheimer’s Disease Assessment Scale cognitive subscale score was significantly improved, but this reverted back to baseline after the washout. After a 12-week trial with modified KD in 21 AD patients, daily function and quality of life improved (148). Changes in cardiovascular risk factors were mostly favorable, and adverse effects were mild in this study.

Therapeutic hyperketonemia was achieved in a patient with Alzheimer’s disease dementia during oral administration of 28.7 grams βHB thrice daily. After 20 months of treatment, improvements in behavior and cognitive and daily activity performance were observed (149).

### Parkinson’s disease

Nutrition plays an important role in risk for developing PD and as modifier during the disease progression (150). High prevalence of insulin resistance is suggested in patients with PD ranging from 50 to 80% and type 2 diabetes is associated with an increased risk of PD (151). Insulin receptors are abundant in the brain, however, concentrated in some areas such as the substantia nigra and basal ganglia. Reduced insulin-mediated glucose uptake was found in newly diagnosed untreated adults with PD (152). Yet, dietary glycemic index is inversely associated with the risk of Parkinson’s disease (153). So, high glycemic index carbohydrates might decrease the risk of Parkinson’s disease (PD) by an insulin-induced increase in brain dopamine. However, epidemiological studies about carbohydrate consumption and PD remain inconclusive.

Mitochondrial dysfunction is a fundamental and complex hallmark in many neurodegenerative disorders, including PD. KB can enhance mitochondrial oxidative phosphorylation and mitochondrial biogenesis in the substantia nigra and bypass complex I deficiency (154, 155). Nutritional supplements coenzyme Q10 and fish oil have also been associated with reduced PD progression (156).

Animal and *in vitro* studies have demonstrated beneficial effects of KB on the course of PD. It was shown that βHB acts *in vitro* as a neuroprotective agent against the toxicity of MPTP, which induces a defect in the mitochondrial complex I of dopaminergic neurons (157). Infusion of βHB in mice treated with the MPTP neurotoxin confers partial protection against dopaminergic neurodegeneration possibly mediated by βHB effects on complex II and by improvement of cellular respiration and ATP production (107). KB also show prevention of synaptic dysfunction induced by mitochondrial respiratory complex inhibitors (Rotenone and 3-nitropropionic acid) in rat brain slides. The protective effects of KB could result from possible antioxidative activity, improved ATP synthesis, and from the effect on the ATP-sensitive potassium channel (KATP) (158, 159).

Human trials conducted being limited, heterogeneous and lacking PD-specific outcomes (160). In a first clinical pilot in 5 patients with PD, improvements in motor score was noticed after 4 weeks of KD (161). A possible (additional) reason for improvement could be the low intake of proteins (8% in this study) which increases the bioavailability of levodopa, a drug used to treat motor symptoms. In a 8-week, randomized, controlled trial to assess the effect of a low-fat vs. ketogenic diet in 38 PD patients, an improvement in motor and non- motor symptoms was found in both groups (162). The ketogenic group however, showed greater improvements in non-motor symptoms. This is an important finding, since treatment with levodopa mainly addresses motor symptoms and does not adequately control many non-motor symptoms. Protein intake was kept at approximately 1 g per kg of body weight per day within each diet group to prevent increased bioavailability of levodopa. In some patients, KD exacerbated the PD tremor and/or rigidity. Perhaps the abrupt increase in fat intake augmented dopamine depletion and/or oxidative stress in the substantia nigra (163). Whether cognitive gains would be maintained upon discontinuation of the KD (or KB supplementation) remains unknown so far.

### Amyotrophic lateral sclerosis

Amyotrophic lateral sclerosis (ALS) is an adult-onset neurodegenerative disorder in which spinal and cortical motor neurons progressively degenerate. Mutations in the gene encoding Cu/Zn superoxide dismutase 1 (*SOD1*) is found in a small percentage of familial ALS. Mutant *SOD1* has been localized in the mitochondria. KD fed *SOD1-G93A* transgenic ALS mice lost 50% of baseline motor performance 25 days later than the disease controls through the gain in mitochondrial energy production (164). In line, administration of caprylic triglyceride, a medium-chain triglyceride, to mutant *SOD1* mice resulted in delayed motor function and better preserved spinal cord motor neuron counts (165).

Within the PatientsLikeMe Community, seven ALS patients reported using ketogenic diets of whom two reported “moderate” effectiveness (166). A small randomized, double-blind trial that compared a high-fat with a high-carbohydrate hypercaloric diet and an isocaloric control diet reported more adverse events during the 6-months high-fat hypercaloric diet, including weight loss (167). Adequate caloric intake is essential in amyotrophic lateral sclerosis and future studies should monitor malnutrition closely (168).

### Multiple sclerosis

Nutritional ketosis has anti-inflammatory properties that can be beneficial to patients with Multiple Sclerosis (MS) (169). In mice experimental autoimmune encephalitis, a mouse model for MS, calorie restriction 3 days a week for 5 weeks was effective in ameliorating symptoms and completely reversed disease progression in a portion of animals (170). KD had more modest effects and did not reverse EAE progression in mice.

In a RCT with 60 relapsing-remitting MS patients, a diet low on carbohydrates (<50 g carbohydrates) for 6 months or a 7 days low caloric intake (10–18% of normal) followed by a Mediterranean diet for 6 months showed improved quality of life compared to regular diet with a mild reduction in expanded disability status scale (EDSS) scores (170). After 6 months on the low carbohydrates diet, serum levels of neurofilament light chain, a biomarker of neuroaxonal damage, were significantly reduced compared with regular diet in patients with relapsing-remitting MS (171). Also the expression of enzymes involved in the biosynthesis of pro-inflammatory eicosanoids was reduced (172). A comparison of 15 patients on a MCT diet, a modified paleolithic diet or usual diet showed only a significant reduction on fatigue scores and maintained cognitive function scores for the modified paleolithic diet compared to the control group (173). A pilot study with twenty subjects and a phase II trial with 65 patients with relapsing MS were enrolled into a 6-month prospective MAD intervention. In these MS patients, KDs were reported to be safe and tolerable. Improvements were seen in body composition, fatigue, depression, quality of life, and neurologic disability. Reduced pro-inflammatory adipokines and elevated anti-inflammatory adipokines were found in the serum (174, 175).

Studies of a KD in neurometabolic degenerative disorders, and more specifically AD and PD consistently demonstrated improved learning and memory. Especially in the prodromal stage of the diseases, nutritional ketosis might delay start of symptoms. Its role in disease progression needs further evaluation. For other neurodegenerative diseases like ALS and MS, the evidence for the therapeutic value of KD or KS are limited, yet promising. In ALS mouse models delayed motor function is observed and in MS anti-inflammatory properties of nutritional ketosis can result in improvements in fatigue, depression, quality of life, and neurologic disability (176).

## Conclusion/future directions

Nutritional ketosis is a well-established strategy for treating epilepsy and has plausible mechanisms for treating neurodegenerative and heart diseases as well. As a performance enhancing strategy, the role of KB supplementation is equivocal, and it might be of added value especially in long duration, low-intensity (ultra) endurance exercise. Both KD and orally administered exogenous KB supplementation elicit widespread physiological changes at both a systemic and cellular level. Unraveling the exact mechanisms for the different nutritional strategies will lead to precision nutrition. International recommendations for the management of children (21) and adults (177) treated with KD have been composed.

## Author contributions

CS and ST wrote and revised the manuscript. Both authors contributed to the article and approved the submitted version.

## Abbreviations

AD: Alzheimer’s disease
ALS: Amyotrophic lateral sclerosis
APOE: Apolipoprotein E
ATP: adenosine triphosphate
βHB: beta-hydroxybutyric acid
FA: fatty acids
GABA: gamma-amino butyric acid
KB: ketone bodies
KD: Ketogenic diet
MAD: Modified Atkins diet
MCT: medium-chain triglyceride
MS: multiple sclerosis
PD: Parkinson’s disease
PEO: progressive external ophtalmoplegia
PPAR: peroxisome proliferator activated receptor
PUFA: polyunsaturated fatty acids
RCT: randomized controlled trial
ROS: reactive oxygen species
SGLT2: sodium-glucose cotransporter-2
UCP: uncoupling proteins
VLCKD: very-low-carbohydrate ketogenic diet.
